# The effect of bee pollen on bone biomechanical strength and trabecular bone histomorphometry in tibia of young Japanese quail (*Coturnix japonica*)

**DOI:** 10.1371/journal.pone.0230240

**Published:** 2020-03-18

**Authors:** Ewa Tomaszewska, Sebastian Knaga, Piotr Dobrowolski, Krzysztof Lamorski, Mirosław Jabłoński, Agnieszka Tomczyk-Warunek, Mohammed Jard Kadhim, Monika Hułas-Stasiak, Grzegorz Borsuk, Siemowit Muszyński

**Affiliations:** 1 Department of Animal Physiology, University of Life Sciences in Lublin, Lublin, Poland; 2 Institute of Biological Basis of Animal Production, University of Life Sciences in Lublin, Lublin, Poland; 3 Department of Comparative Anatomy and Anthropology, Maria Curie-Skłodowska University, Lublin, Poland; 4 Bohdan Dobrzański Institute of Agrophysics of the Polish Academy of Sciences, Lublin, Poland; 5 Chair and Department of Rehabilitation and Orthopaedics, Medical University in Lublin, Lublin, Poland; 6 Department of Animal Production Techniques, Al- Furat Al- Awsat Technical University, Babylon, Iraq; 7 Department of Biophysics, University of Life Sciences in Lublin, Lublin, Poland; University of Illinois, UNITED STATES

## Abstract

It has been demonstrated in numerous studies that bee pollen supplementation shows numerous positive effects on health. However, its impact on bones is largely unknown. The purpose of this study was to investigate the effect of bee pollen supplementation on the tibia biomechanical properties and bone morphometric measures using Japanese quail as an animal model. The experiment was arranged in a 2x2x2 factorial design, with sex, quail line (meat-type or egg-lying type), and bee pollen inclusion (0 or 10 g/kg of feed) as factors. The quails were one-day-old at the beginning of the experiment, they were euthanized after 42 days. Our study showed for the first time unfavorable effects of bee pollen on bones properties. Bee pollen supplementation negatively affected bone structure, irrespective of quails’ sex or line type. Bone length (P < 0.001), weight (P < 0.01), and mean relative wall thickness (P < 0.01) and mineralization (P < 0.05) were reduced by bee pollen treatment. For female quails, irrespective of line type, the decrease of yield load (P < 0.001), ultimate load (P < 0.01), yield stress (P < 0.001) and ultimate stress (P < 0.05) was noted. Analysis of growth plate in bone metaphysis showed that bee pollen supplementation slowed the process of bone maturation irrespective of sex (P < 0.05). On contrary, dietary bee pollen positively affected bone homeostasis of trabecular bone in bone metaphysis as bone mineral density increased in experimental groups (P < 0.05). In males, this was the result of the increase of trabecular thickness (P < 0.01), in females due to the reduction of trabecular space (P < 0.001). In conclusion, our results demonstrate that bee pollen (1.0%, 10 g/kg of feed) supplementation caused significant negative effects on the mechanical endurance of the tibia of quails, while showed beneficial effects on trabecular bone histomorphometry.

## Introduction

Bee pollen is a mixture of flower pollen agglutinated by nectar and honeybee salivary enzymes. The major components of bee pollen are proteins, essential amino acids, reducing sugars, lipids, nucleic acids, minerals, vitamins, as well as enzymes and co-enzymes necessary for good digestion [1]. This natural product is considered a health food, as it exhibit a wide range of therapeutic properties, including: antioxidant, antimicrobial, antifungal, hepatoprotective, and anti-inflammatory activities; for detailed review see [2]. The very recent review lists numerous positive effects of bee pollen supplementation on poultry, showing that dietary bee pollen inclusion may exert positive immunomodulatory effects, antioxidants effects, anti-inflammatory effects, improve blood profile and biochemistry, improve cardiac, liver and kidney functions [3].

However, in available literature review, there is little information about the influence of bee pollen supplementation on bone development and functionality. There are some *in vitro* studies suggesting that bee pollen has a stimulatory effect on bone formation and an inhibitory effect on bone resorption [4–6], however its impact on biomechanical properties and bone microstructure in *in vivo* studies has been analyzed to a limited extent [7, 8].

The Japanese quail (*Coturnix japonica*) has been extensively used as an experimental model across various disciplines within the life sciences for decades [9, 10]. As an animal avian model, Japanese quail is often used in studies of the toxicology of chemical compounds and the effects of environmental endocrine disruptors or in examining the physiological processes in birds [11–13]. Quails are also used as an animal model for studying bone formation and development, both in pre-hatch and post-hatch studies [14–21].

Therefore, the objective of this study was to investigate the effect of administration of bee pollen on selected biomechanical characteristics and histological structure of tibia using both young males and females of two lines of Japanese quails, a meat-type line and egg-lying line, as an animal model.

## Material and methods

The experimental procedures used throughout this study were approved by the Local Ethics Committee on Animal Experimentation of University of Life Sciences in Lublin, Poland (8/2015). This study was carried out in strict accordance with the recommendations of the National Ethic Commission (Poland).

### Animals and experimental feeds

The study was carried out on healthy male and female Japanese quails of standard (S-33, egg-lying type, n = 144) and pharaoh (F-33, meat-type, n = 144) lines from the breading flocks of the University of Life Sciences in Lublin, Poland. Hatched quail chicks were randomly allocated to two dietary treatments (n = 72 from each line); a control, fed basal-diet without bee pollen, or a bee pollen group, fed experimental diet including 1.0% of bee pollen (10 g bee pollen/kg) (Table 1). All feeds used in the experiment were prepared in industrial feed mixing facility (Agropol, Motycz, Poland) in accordance with the prepared guidelines. The basal diet was formulated to meet or exceed nutrient requirements for quails [22, 23]. The multiflora pollen was obtained from a commercial firm in Poland (Apis Apiculture Cooperative, Lublin, Poland). Pollen baskets are supplied to the Apis Apiculture Cooperative by several leading pollen producers in the Lublin region, Poland. The batch of the pollen baskets used in the experiment was supplied by a beekeeper, who rears *Apis mellifera carnica* bees in mobile apiary consisting of 300 bee colonies kept in box hives. He uses top-mounted pollen traps so that pollen falling into the trap draw can be dried by the heat generate by the bee colony, thereby preventing moulding. Bee pollen was collected every day during the harvest and dried at a temperature of 37°C with air circulation in pollen driers. It was dried to water content of approx. 5%. Next, it was packed into paper bags, which were transferred to tightly closed zip bags, which prevented absorption of moisture from the air. The beekeeper has several pollen reward sites, where he transports his bee colonies. The pollen used in the experiment originated from the Sobibór Landscape Park (51°29'04.1"N; 23°34'53.8"E) on the river Bug. The bees used multi-flower pollen reward in May 2016 dominated by common dandelion (*Taraxacum officinale*) pollen. The nutrient composition of the diets and bee pollen was analyzed according to AOAC procedures [24] in the Central Laboratory of Agroecology of University of Life Sciences in Lublin (Tables 1 and 2). Crude protein, crude fibre and crude fiber in the diets were determined according to method 954.01, 920.39, and 978.10 respectively. The total phosphorus was determined colorimetrically (method 965.17), whereas the content of Ca was determined using the FAAS technique (method 968.08) [24]. The content of crude protein, crude fat, crude fibre in bee pollen was determined according to method 928.08, 991.36, and 991.43 respectively. The amino acid composition was determined by amino acid analyzer (method 994.12); macro- and microelements were determined using ICP-ES (984.27 method), while the carbohydrates composition was determined by gas chromatography (method 971.18) [24]. The bee pollen dose was chosen in the preliminary study performed on male and female quails (S-33 line) supplemented with 0.5%, 1.0%, 3.0% or 5.0% of bee pollen, with the selection criterion of the highest bee pollen dose which did not influence body weight gain [25]. The diet and water were available *ad libitum* from day 1. The birds were housed in environmentally controlled poultry house in cages allowing at least 0.042 m^2^ of space for each bird during the rearing period [26]. The rearing temperature was gradually decreased from 38 to 34°C in the first week, from 33 to 28°C in the second week, and from 27 to 22°C in the third week. Afterward, it was maintained between 18 and 20°C [27, 28]. Each of the experimental group was randomly subdivided into 6 subgroups (replicates), comprising 12 birds each. After being separated at three weeks of age according to sex by qualified technician basing on the colour of the plumage and through cloacal gland inspection, female and male quails in each group were transferred to separate cages. Therefore, in the experiment a 2x2x2 factorial arrangement was employed, with quail line (standard or pharaoh), sex (male or female) and bee pollen inclusion (0 or 10 g/kg of feed) as factors with 6 replicate cages per each experimental group (48 cages in total). Each replication cage contained 6 birds with the expectation of 11 cages containing 5 birds, because it was not possible to select the target number of individuals of one sex in these replications. The experiment lasted until 6 weeks of age. At the end of the experiment, 6 birds (one per replicate cage) were randomly selected from each group, fasted for 8 h, weighed and slaughtered by decapitation after mechanical stunning (48 birds in total, 6 individuals from each of 8 experimental groups). After the slaughter, both tibiae were dissected and kept frozen at -25°C until further examination. In subsequent stages of analyses, right tibiae were subjected to strength test, determination of bone weight, length, volumetric density and bone ash percentage, while left tibiae were earmarked for geometric measurements and histomorphometric analysis. Before all analyses, the frozen bones were thawed overnight in the laboratory at 5°C.

**Table 1:** Composition and nutritive value of feed mixtures for Japanese quail.

Item	Control diet	Bee pollen supplemented diet
*Ingredient*, *g/kg of feed*		
Maize	150	150
Wheat	179.4	179.4
Triticale	250	250
Oats	50	50
Soybean meal, 48% CP	98.5	88.5
Bee pollen	0	10
Rapeseed meal	35	35
Sunflower meal	100	100
Soybean oil	12	12
Monocalcium phosphate	3.5	3.5
Mel stern	47	47
Sodium bicarbonate	1.5	1.5
Phyzyme	0.06	0.06
Sodium chloride	2.7	2.7
DL-methionine 99%	0.5	0.5
L-lysine HCl	2.2	2.2
Ronozyme	0.14	0.14
L-threonine 99%	0.5	0.5
Fat protein concentrate	10	10
Calcium granules	52	52
Pigment	5	5
*Calculated nutritional value 1 kg of feed*, *MJ/kg*		
Metabolizable energy	10.88	10.88
*Analyzed composition of the basal feed mixtures*, *g/kg*		
Crude protein	211.5	207.4
Crude fat	51.3	48.5
Crude fibre	45.7	52.8
Ca	20.5	30.1
Total P	6.1	5.4

**Table 2:** Chemical composition of bee pollen used in the experiment.

Item	Content
*Main components*, *%*	
Protein	27.4
Crude fibre	6.82
Fat	6.30
*Carbohydrates*, *g/100g*	
Sucrose	4.49
Glucose	12.6
Fructose	15.3
Sorbitol	1.08
*Macroelements*, *g/kg*	
Ca	1.54
Mg	1.04
K	4.36
*Microelements*, *mg/kg*	
Na	125
Cu	8.80
Fe	25.6
Mn	23.8
Zn	30.7
*Amino acids*, *mg/g*	
Asp	25.1
Thr	11.9
Ser	13.1
Glu	27.3
Pro	19.9
Gly	11.6
Ala	13.5
Val	12.5
Ile	9.9
Leu	16.8
Tyr	6.12
Phe	11.4
His	6.87
Lys	16.7
Arg	11.9

### Bone analysis and mechanical test

Tibiae weight, length, and the Seedor index (bone weight to length ratio) as the indicator of whole bone density were determined. To examine the mechanical properties of bones a three-point bending test was performed on a Zwick Z010 universal testing machine (Zwick-Roell GmbH & Co., Ulm, Germany) [29]. The bones were loaded in the midpoint of the bone diaphysis in the cranial-caudal plane with the loading rate 5 mm/min. Bone structural traits (yield load, ultimate load, elastic energy, work to fracture, and stiffness) were determined from recorded force-displacement curves using Origin 2016 software (OriginLab, Northampton, MA, USA) [30]. The measurement of bone volumetric density (BVD) was performed on the sheared tibia pieces with an AccuPyc 1330 helium gas pycnometer (Micromeritics, Inc., Norcross, GA, USA) [31]. Finally, the bones were defatted, weighted, and ashed in a muffle furnace at 500 °C for 24 h to determine the ash percentage.

### Bone diaphysis geometry and bone material traits

Left tibiae were cut across in the midpoint of the bone diaphysis with a diamond bandsaw (MBS 240/E, Proxxon GmbH, Foehren, Germany). Horizontal (medial-lateral plane) and vertical (cranial-caudal plane) external and internal diameters of the bone diaphysis cross-section were measured with a digital caliper. On the basis of these measurements the following geometric traits of bone diaphysis were calculated: mean relative wall thickness (MRWT), cortical cross-sectional area and cross-sectional moment of inertia (CSMI) [30]. Whole-bone material traits (Young’s modulus, yield stress, yield strain, ultimate stress, ultimate strain, and toughness modulus) were calculated on the basis of bone structural traits determined during a three-point bending test and measured bone diaphysis cross-sectional geometry using engineering beam-theory equations [30].

### Histomorphometrical analysis

After geometric measurements, the proximal end of left tibia was cut off and fixed in buffered formaldehyde. Formalin-fixed samples were dehydrated with Ottix Plus, cleared with Ottix Shaper solvent substitutes (DiaPath, Martinengo, Italy), embedded in paraffin, and finally sectioned with a microtome in 4-μm slices. Two microscopic slides were prepared. The sections on one slide were stained with the Picrosirius red (PSR) staining for the morphology of the trabecular bone. Second slide was stained with Goldner’s trichrome staining to assess the thickness of the growth plate cartilage. Stained slides were observed in normal (Goldner) and polarized (PSR) light by light microscopy (CX43, Olympus, Tokyo, Japan). Ten microscopic fields per bird were recorded using a high-resolution CDD camera (SC50, Olympus, Tokyo, Japan). PSR images were analysed using ImageJ software (NIH, Bethesda, MD, USA) for assessment of morphometric traits of trabecular bone: relative bone volume (BV/TV), mean trabecular thickness (Tb.Th mean), maximal trabecular thickness (Tb.Th mean), mean trabecular separation (Tb.Sp mean), maximal trabecular separation (Tb.Sp max), and trabecular number (Tb.N) [32].

### Statistical analysis

Data of the experiment were subjected to statistical analysis using 3-way analysis of variance (ANOVA) of general linear model (Statistica 13, TIBCO Software Inc., Palo Alto, CA, USA) according to a (2×2×2) factorial arrangement of treatments with the model including diet, line, and sex as the main factors and their 2- or 3-way interactions. Before testing for group differences, data were checked for normal distribution (Shapiro-Wilk test) and homogeneity of variance (Levene test). One replicate cage constituted an experimental unit and the values presented in the tables are means with standard deviations (SD) and pooled standard error of the means (SEM). If a significant effect was detected, differences between treatments or main effects were separated by Duncan’s multiple range test. Significant differences among treatments were determined for P < 0.05.

## Results

### Rearing results

Bird’s body weight at the end of rearing period at the age of 6 weeks was not affected by dietary bee pollen inclusion (Table 3). However, body weight was dependent on both quails’ sex and line (P < 0.001). Females were heavier than males (139 g and 125 g, respectively), and meat-type F-33 quails were heavier than those of egg-lying S-33 line (139 g and 124 g, respectively).

**Table 3:** The effect of bee pollen supplementation on body weight and bone morphology in 6 weeks old Japanese quails.

Factors			Body weight, g	Bone mass, g	Bone length, mm	The Seedor index, mg/mm	MRWT, —	Cross- sectional area, mm^2^	CSMI, mm^4^	BVD, g/cm^3^	Ash, %
line	sex	bee pollen									
pharaoh	m	−	133 ±5	0.429 ±0.029	38.3 ±1.3	11.2 ±0.6	3.66 ±0.61	3.77 ±0.38	2.18 ±0.38	1.81 ±0.07	39.7 ±1.1
	m	+	135 ±6	0.360 ±0.056	36.3 ±2.2	9.8 ±1.0	2.37 ±0.25	3.16 ±0.49	1.75 ±0.21	2.20 ±0.22	38.8 ±1.5
	f	−	145 ±12	0.443 ±0.044	39.6 ±0.7	11.2 ±1.2	3.22 ±0.51	3.64 ±0.67	2.20 ±0.39	1.98 ±0.13	42.3 ±2.1
	f	+	143 ±13	0.361 ±0.078	35.2 ±2.8	10.2 ±1.5	2.07 ±0.49	3.43 ±0.42	1.39 ±0.31	2.78 ±0.25	38.0 ±2.3
standard	m	−	116 ±5	0.380 ±0.029	37.3 ±1.6	10.2 ±0.8	3.63 ±1.26	3.30 ±0.44	1.81 ±0.42	1.98 ±0.21	39.7 ±2.2
	m	+	114 ±6	0.320 ±0.077	34.7 ±3.4	9.1 ±1.4	2.94 ±0.83	3.32 ±0.35	1.69 ±0.45	2.44 ±0.34	40.1 ±3.1
	f	−	135 ±11	0.417 ±0.053	38.6 ±0.73	10.8 ±1.8	3.21 ±0.43	3.85 ±0.40	1.98 ±0.23	2.28 ±0.10	51.6 ±5.5
	f	+	133 ±9	0.367 ±0.072	37.3 ±1.33	9.8 ±1.6	3.00 ±1.06	3.75 ±0.07	1.84 ±0.47	2.39 ±0.42	47.1 ±6.7
Pooled SEM			4	0.026	0.9	0.5	0.34	0.20	0.17	0.15	1.6
*Main factors*											
pharaoh			139	0.398	37.3	10.6	2.83	3.50	1.88	2.19	39.7
standard			124	0.371	37.0	9.98	3.20	3.55	1.83	2.27	44.6
	m		125	0.373	36.7	10.1	3.15	3.39	1.86	2.11	39.6
	f		139	0.397	37.7	10.5	2.88	3.67	1.86	2.36	44.7
		−	132	0.417	38.5	10.8	3.43	3.64	2.04	2.02	43.3
		+	131	0.352	35.9	9.7	2.60	3.42	1.67	2.45	41.0
*Main effects and interactions*											
line (L)			***	NS	NS	NS	NS	NS	NS	NS	***
sex (S)			***	NS	NS	NS	NS	*	NS	*	***
bee pollen (BP)			NS	**	***	**	**	NS	**	***	*
L x S			NS	NS	NS	NS	NS	NS	NS	NS	***
S x BP			NS	NS	NS	NS	NS	NS	NS	NS	NS
L x BP			NS	NS	NS	NS	NS	NS	*	NS	NS
L x S x BP			NS	NS	NS	NS	NS	NS	NS	NS	NS

Data given are mean (n = 6) with corresponding standard deviations; p-values: NS—not significant;

* P < 0.05;

** P < 0.01;

*** P < 0.001.

m—male; f—female; SEM—standard error of the means.

MRWT—mean relative wall thickness; CSMI—cross-sectional moment of inertia; BVD—bone volumetric density.

### Bone osteometry and densitometry

Tibia osteometric and densitometric data are presented in Table 3. Bee pollen supplementation resulted in reduction of bone mass (P < 0.01), length (P < 0.001) the Seedor index (P < 0.01), and MRWT of bone diaphysis (P < 0.01). On the contrary, bone volumetric density increased in bee pollen-supplemented groups (P < 0.001). Bone volumetric density was also dependent on quails’ sex, and more dense tibias were observed in females (P < 0.05).

There was a significant interaction between line and bee pollen supplementation on CSMI (P < 0.05). The effect of pollen addition was found to be significant only for pharaoh line for which the decrease of CSMI was observed. Ash percentage depended on both bee pollen supplementation (P < 0.05) and an interaction between line and sex (P < 0.001). Bee pollen supplementation decreased bone ash percentage, irrespective of sex or line (P < 0.05).

### Bone biomechanical properties

The results of analysis of tibia mechanical properties are summarized in Table 4. Numerous bone structural and material properties were line-dependent. Quails of S-33 standard line were characterized with greater ultimate load (P < 0.05), stiffness (P < 0.05), Young’s modulus (P < 0.05), and ultimate stress (P < 0.01) when compared to F-33 pharaoh line. When bee pollen was added to diet, a significant increase of ultimate strain (P < 0.01) and toughness modulus (P < 0.05) was observed. There were also significant interactions between bee pollen supplementation and quail’s sex. The highest values of yield load (P < 0.001), ultimate load (P < 0.01), stiffness (P < 0.01), elastic energy (P < 0.05), yield stress (P < 0.001), and ultimate stress (P < 0.05) were observed for females fed control diet and bee pollen supplementation decreased the values of these parameters. The analysis of the effects of other interactions showed that the highest values of yield load were observed for females of standard line (P < 0.05), while the greatest yield load was noted for birds of standard line fed without bee pollen supplementation (P < 0.01). There was no effect of line type, sex, or bee pollen inclusion on work to fracture or yield strain.

**Table 4:** The effect of bee pollen supplementation on biomechanical tibia properties in 6 weeks old Japanese quails.

Factors			Yield load, N	Ultimate load, N	Stiffness, N/mm	Elastic energy, mJ	Work to fracture, mJ	Young’s modulus, GPa	Yield strain, %	Ultimate strain, %	Yield stress, MPa	Ultimate stress, MPa	Toughness modulus, mJ/mm^3^
line	sex	bee pollen											
pharaoh	m	−	22.7 ±3.9	25.1 ±4.7	96.2 ±23.1	2.50 ±1.13	4.26 ±2.09	5.70 ±1.58	1.01 ±0.45	1.50 ±0.58	52.0 ±7.6	57.6 ±8.9	0.66 ±0.32
	m	+	25.3 ±3.6	30.6 ±3.5	77.2 ±14.4	3.58 ±0.62	9.64 ±2.62	5.73 ±0.49	1.21 ±0.11	2.59 ±0.59	69.5 ±9.8	84.4 ±11.8	1.76 ±0.60
	f	−	32.0 ±4.3	37.7 ±7.3	122.5 ±29.7	3.97 ±1.15	7.42 ±2.67	6.06 ±1.19	1.25 ±0.32	1.91 ±0.53	73.2 ±10.2	86.5 ±18.4	1.17 ±0.44
	f	+	19.8 ±3.8	24.7 ±5.0	50.4 ±21.1	3.13 ±1.21	8.46 ±2.53	5.61 ±1.23	1.17 ±0.42	2.57 ±0.41	61.8 ±6.9	77.0 ±8.8	1.56 ±0.27
standard	m	−	25.0 ±2.9	32.5 ±7.7	105.8 ±19.6	2.87 ±1.10	5.65 ±2.98	6.76 ±2.21	1.10 ±0.40	1.67 ±0.71	68.0 ±10.0	89.3 ±17.8	1.01 ±0.57
	m	+	23.3 ±2.7	29.5 ±4.6	81.9 ±8.5	2.75 ±1.15	7.98 ±3.18	7.22 ±2.21	1.01 ±0.40	2.19 ±0.77	66.9 ±8.6	84.7 ±19.8	1.44 ±0.57
	f	−	38.0 ±7.0	43.7 ±9.7	140.0 ±18.4	4.90 ±1.67	9.61 ±4.84	7.54 ±1.09	1.25 ±0.30	1.94 ±0.79	92.0 ±10.2	105.3 ±14.1	1.50 ±0.82
	f	+	25.4 ±2.5	32.8 ±4.3	91.6 ±32.5	3.06 ±0.76	9.23 ±4.36	6.25 ±1.22	1.09 ±0.18	2.40 ±0.42	66.6 ±8.9	85.7 ±10.8	1.52 ±0.80
Pooled SEM			1.8	2.8	9.9	0.51	1.60	0.67	0.15	0.33	4.1	7.3	0.26
*Main factors*													
pharaoh			24.9	29.5	86.6	3.30	7.44	5.77	1.16	2.14	64.1	76.3	1.29
standard			27.9	34.6	104.8	3.40	8.12	6.94	1.11	2.05	73.1	91.3	1.38
	m		24.1	29.4	90.3	2.93	6.88	6.35	1.08	2.00	63.9	79.0	1.22
	f		28.8	34.7	101.1	3.77	8.68	6.36	1.19	2.21	73.4	88.6	1.44
		−	29.4	34.8	116.1	3.56	6.74	6.51	1.15	1.76	71.3	84.7	1.08
		+	23.4	29.4	75.3	3.13	8.83	6.20	1.12	2.44	66.0	82.9	1.57
*Main effects and interactions*													
line (L)			*	*	*	NS	NS	*	NS	NS	**	**	NS
sex (S)			***	*	NS	*	NS	NS	NS	NS	**	NS	NS
bee pollen (BP)			***	**	***	NS	NS	NS	NS	**	NS	NS	*
L x S			*	NS	NS	NS	NS	NS	NS	NS	NS	NS	NS
S x BP			***	**	**	*	NS	NS	NS	NS	***	*	NS
L x BP			NS	NS	NS	NS	NS	NS	NS	NS	**	NS	NS
L x S x BP			NS	NS	NS	NS	NS	NS	NS	NS	NS	NS	NS

Data given are mean (n = 6) with corresponding standard deviations; p-values: NS—not significant;

* P < 0.05;

** P < 0.01;

*** P < 0.001.

m—male; f—female; SEM—standard error of the means.

### Histomorphometry of trabecular bone

**Fig 1:**
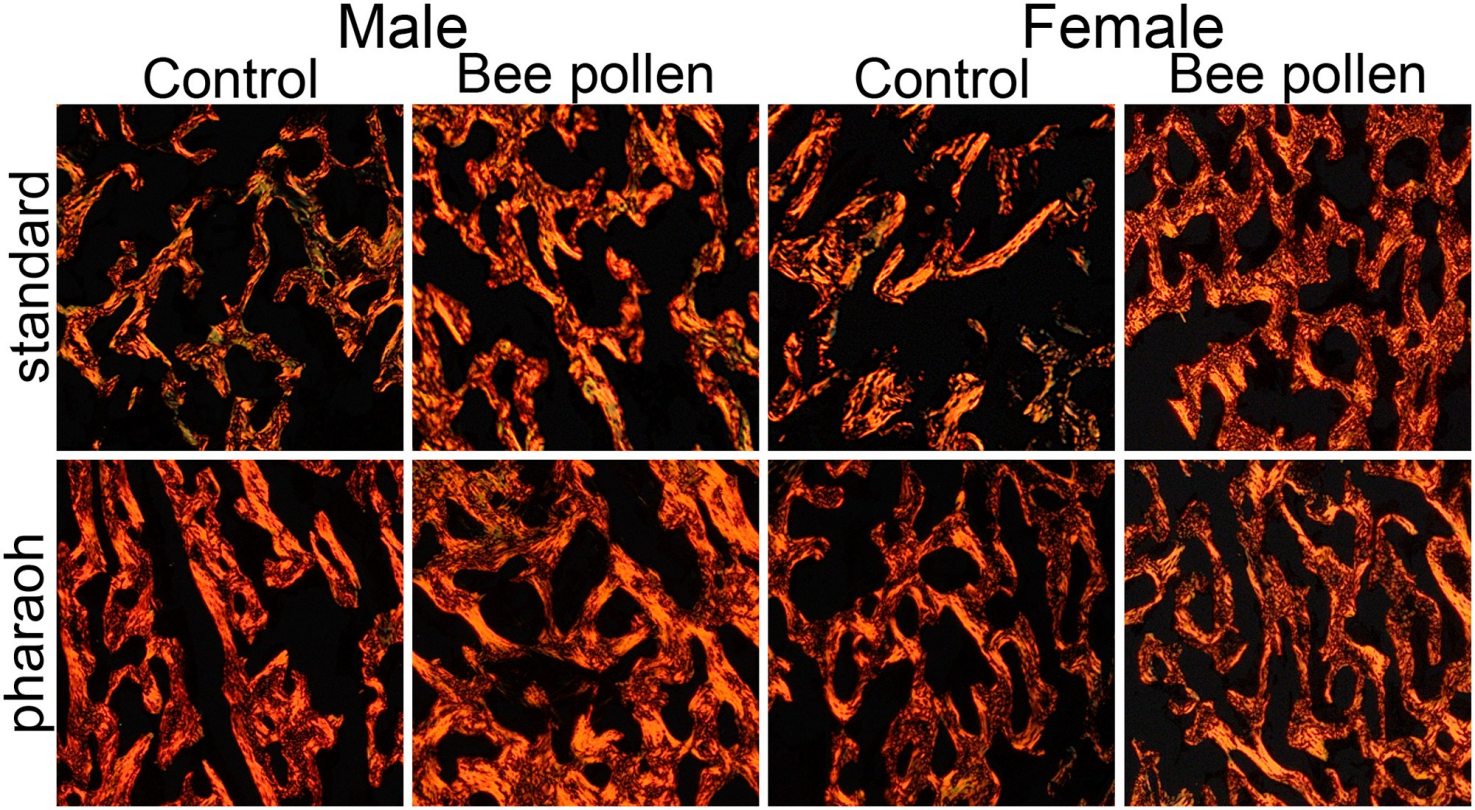
Representative images of PSR stained sections of trabecular bone of tibia metaphysis of 6-weeks-old male and female Japanese quails of S-33 standard (egg-lying type) and F-33 pharaoh (meat-type) lines.

Table 5 shows the results of analysis of growth plate cartilage thickness and trabecular bone histomorphometry. Bee pollen supplementation significantly increased growth plate thickness in both male and female quails, irrespective of the line type (P < 0.05). In trabecular bone, bee pollen supplementation significantly increased bone volume density (BV/TV, P < 0.05) and maximal trabecular thickness (P < 0.01), irrespective for birds’ sex or line type (Fig 1). For the other histomporhometrical indices, the effect of bee pollen supplementation was sex-depended, as indicated by numerous interactions. In males, bee pollen supplementation increased mean trabecular thickness (P < 0.01) and decreased trabecular number (P < 0.001), while in females the decrease of mean trabecular space was observed (P < 0.01).

**Table 5:** The effect of bee pollen supplementation on growth plate thickness and on trabecular bone morphology in 6 weeks old Japanese quails.

Factors			Growth plate, μm	BV/TV %	Tb.Th mean, μm	Tb.Th max, μm	Tb.Sp mean, μm	Tb.Sp max, μm	Tb.N, μm
line	sex	bee pollen							
pharaoh	m	−	254 ±63	50.0 ±7.1	30.4 ±8.0	76.4 ±19.6	54.1 ±9.4	155 ±30	17.0 ±2.5
	m	+	430 ±140	51.1 ±10.4	41.8 ±9.5	85.8 ±23.5	64.0 ±15.8	172 ±38	12.3 ±1.5
	f	−	617 ±115	48.7 ±7.2	35.8 ±10.2	77.9 ±28.5	70.4 ±20.1	167 ±57	14.3 ±2.7
	f	+	802 ±113	51.7 ±6.7	35.4 ±10.4	83.6 ±23.3	53.0 ±11.2	154 ±62	15.4 ±3.3
standard	m	−	484 ±69	40.5 ±6.4	25.1 ±5.0	51.4 ±9.0	58.8 ±13.4	129 ±33	16.4 ±2.5
	m	+	611 ±115	51.1 ±10.2	41.8 ±8.6	85.8 ±20.5	64.0 ±12.6	172 ±43	12.3 ±2.1
	f	−	506 ±113	47.4 ±5.8	32.2 ±7.8	69.8 ±17.4	60.3 ±15.6	161 ±50	15.3 ±3.5
	f	+	432 ±133	48.3 ±5.6	34.5 ±6.7	78.5 ±12.7	53.3 ±8.3	137 ±54	14.3 ±2.3
Pooled SEM			57	2.6	2.9	7.1	4.9	16	0.8
*Main factors*									
pharaoh			526	50.4	35.8	80.9	60.4	162	14.7
standard			508	46.8	33.4	71.4	59.1	150	14.6
	m		445	48.2	34.8	74.9	60.2	157	14.5
	f		590	49.0	34.5	77.5	59.3	155	14.8
		−	466	46.7	30.9	68.9	60.9	153	15.8
		+	569	50.5	38.3	83.5	58.6	159	13.6
*Main effects and interactions*									
line (L)			0.658	NS	NS	NS	NS	NS	NS
sex (S)			***	NS	NS	NS	NS	NS	NS
bee pollen (BP)			*	*	***	**	NS	NS	***
L x S			***	NS	NS	NS	NS	NS	NS
S x BP			NS	NS	**	NS	**	*	***
L x BP			NS	NS	NS	NS	NS	NS	NS
L x S x BP			NS	NS	NS	NS	NS	NS	NS

Data given are mean (n = 6) with corresponding standard deviations; p-values: NS—not significant;

* P < 0.05;

** P < 0.01;

*** P < 0.001.

m—male; f—female; SEM—standard error of the means.

BV/TV—relative bone volume; Tb.Th—trabecular thickness; Tb.Sp—trabecular separation; Tb.N—trabecular number.

## Discussion

In the present study, the effects of bee pollen supplementation on bone morphology, mechanical properties and microstructure in tibia, using the quail as an animal model were investigated. Female quail began to lay eggs from ca. 40 days of age and are in full production by 50 [33]. Therefore, to avoid the effect of egg production on bone metabolism and formation of medullary bone, the study was performed on quails that did not lay eggs.

In quails, females are heavier than males and birds from meat-type lines and heavier that others [34, 35]. This was observed in our study. Despite the differences in body weight, no effect of sex on tibia length was noted, which was also previously reported [33]. However, bone diaphysis cross-sectional area was greater in females than in males. As a result, the bones of females were denser (bone volumetric density) and more mineralized (bone ash percentage). This result is also in agreement with that reported by Nishimura et al., [33], who showed that in quails female bone densities exceed the ones noted for males. The sexual differences in the mineralization, volumetric density and bone diaphysis cross-sectional area indicate positive adaptation of bone structure toward supporting the increased body masses in females [36].

There was also no effect of bee pollen supplementation on quails’ body weight. However, as indicated earlier, we intentionally used in our study the bee pollen dose that did not affect body weight gain in the preliminary study [25]. Unexpectedly, despite the lack of differences in body weight, 1.0% bee pollen supplementation negatively affected bone structure and mineralization in 6 weeks old quails, irrespective of quails’ sex or line type. Bone length, weight, and mean relative wall thickness were all reduced in bee pollen supplemented groups. Only bone diaphysis cross-sectional area did not differ between supplemented and un-supplemented groups. This is an interesting result. Whole bone morphology is an important determinant of skeletal endurance. In tibia, bone diaphysis geometry is critical for the ability to counteract the loads and stresses associated with body weight [37, 38]. Therefore, although there was a change in bone size, bone diaphysis cross-sectional area remained unchanged because the weight of the birds did not differ and the bones were subjected to the same loading.

Our results seem to be in contradiction to previous studies performed on broiler chickens, which show no effect of dietary bee pollen inclusion at the concentration of 0.5% or 1.0% of feed on tibia osteometric traits and mineralization in broilers at 42 days of age [7, 39]. However, in these papers there is a lack of information whether bee pollen supplementation influenced the body weight of chickens. Therefore, it is possible, that chickens from supplemented and un-supplemented groups were characterized by similar osteometric traits of the bones at different body weights. Additionally, an experiment performed on different animal model (rat) partially supports our results, showing that while 90-days long supplementation of bee pollen at the concentration of 0.75% of feed has no significant effect on body weight and femoral length, femora weights were significantly decreased in female rats fed diet supplemented with bee pollen [40].

Performed bending test showed that generally quails of egg-lying line were characterized with stronger bones than those of meat-type line, irrespective of the sex. This result is not surprising, as meat-type poultry lines (broilers) have been selected for rapid growth and are characterized by bones with a lower degree of calcification and higher porosity, which make their bones intrinsically weak [41]. When female quails, irrespective of line type, were supplemented with bee pollen at the concentration of 1.0% of feed the decrease of bone mechanical strength was noted. This was observed for almost all determined bone biomechanical structural traits. Only the work to fracture, representing brittleness, did not differ between un-supplemented and supplemented groups. Especially, the bones of female quails in bee pollen supplemented group were characterized with decreased values of yield and ultimate load and were significantly less rigid, as indicated by reduced bone stiffness.

While bone material properties are better traits than raw bone breaking strength or stiffness in measuring the effect of treatment on bone strength, as they can correct for bone size, the negative effect of 1.0% bee pollen supplementation on bone in female quails was also evident when bone material properties were analyzed. Reduced values of yield and ultimate stresses, which are a measure of the internal resistance of a material to deformity, indicate that bones of female quails receiving 1.0% of bee pollen in feed were able to withstand lower stresses before plastic deformation and fracture. Similarly, much greater fracture deflection (in terms of ultimate strain) and toughness modulus suggest that bones of both male and female quails supplemented with bee pollen were not only the weakest, but also the softest and have the greatest susceptibility to plastic deformation.

The data of bones’ ash measurement indicate that bee pollen-related changes in mechanical properties of bones and their “plastic-like” nature at least partially resultes from their reduced mineralization. In fact, it was expected that the bones of birds fed diets containing bee pollen would have higher mineral content. Bee pollen is rich in vitamin D and amino acids (e.g. Lys, Asp, Glu, Arg; Table 2) which improve calcium absorption. Therefore, bee pollen supplementation should enhance minerals absorption, and subsequently their deposition in the bones. Indeed, Yamaguchi et al. [5] reported higher concentration of Ca in femoral bones of rats after *per os* administration of bee pollen extract (5 or 10 mg/100g body weight).

Bone geometry, weight or mineralization are not only indices for evaluating the morphological changes in bones after dietary treatment [42, 43]. Also bone metaphysis microarchitecture can be an indicator of bone functionality, as bone longitudinal growth occurs as a consequence of conversion of cartilage to mineralized bone within the metaphysis [44, 45]. Despite being 6 weeks old, our quails still seem to have developing bones, irrespective of line type, sex or bee pollen supplementation. In mature bones, the growth plate disappears completely, which in proximal epiphyses of tibia in quails takes place around 6 weeks of age, although in males the process finishes earlier [33, 46]. These findings are partially consistent with our observations, as growth plate cartilages were thinner in males. Supplementation with 1.0% of bee pollen slowed down the process of bone maturation. Interestingly, the thickest growth plate cartilage was observed in tibiae of bee pollen supplemented females from meat-type line, which were characterized by the one of the lowest mechanical strength and mineralization. The deceleration of bone maturation can be associated with the activity of growth factors involved in bone growth such as osteocalcin, insulin-like growth factor I (IGF-I) and steroid hormones [16, 21, 47–49]. There is a study suggesting that bee pollen can act on bone maturation through modulation of the activity of bone growth factors, as Kolesárová et al. [50] report increase in progesterone and estradiol production and decrease in the secretion of IGF-I in female rats after bee pollen supplementation at the dose of 0.5% of feed for 90 days. The mechanisms by which bee pollen influences closure of epiphyseal growth plate and bone maturation should be examined in further studies in more detailed manner.

However, in contrast to all above negative effects, bee pollen supplementation positively affected bone homeostasis of trabecular bone in bone metaphysis as bone mineral density (in terms of BV/TV) increased in bee pollen supplemented groups (Fig 1). In males, this resulted from the increase of trabecular thickness, which was sufficient to compensate for the reduction of the trabecular number. In females the increase of BV/TV was gained mainly due to the reduction of trabecular space. The changes observed in females are very positive in terms of trabecular bone biomechanical strength, as trabecular space is coupled with the trabecular connectivity inside a bone, which contributes more to the bone’s strength than the trabecular thickness [51, 52]. A positive effect on mineralization of trabecular bone was previously reported by Yamaguchi et al. [5], who showed that male rats fed bee pollen (5 or 10 mg/100g body weight) had higher calcium in femoral metaphyseal tissues in comparison to those from the un-supplemented group. Besides calcium, other minerals are present in bee pollen (Table 2). Zinc plays important roles in bone metabolism as a catalyst of many enzymes that affect mineralization, such as bone alkaline phosphatase [53–56]. While alkaline phosphatase activity was not measured in the present study, Yamaguchi et al. [5] showed that dietary bee pollen can promote alkaline phosphatase activity in metaphyseal tissues of bones, which, with increased mineralization, point to enhanced remodeling of trabecular bone. As discussed earlier, our analysis showed that bones of female quails were weaker after 1.0% bee pollen supplementation. However, observed improvement of trabecular bone morphology with previously reported by Kolesárová et al. [50] increase in progesterone and estradiol production observed in mature female rats supplemented with bee pollen (0.3 and 0.5% in feed) suggest that bee pollen might have anti-osteopenic properties, exerting a positive effect on morphological characteristics of trabecular bone in mature females. Yet, without systematic research both on female quail during the lying period and mature mammalian animal model it can be only speculated.

## Conclusions

No study has been published that shows the effects of dietary bee pollen supplementation on bone properties in a such detailed manner in *in vivo* studies. In our study conducted on Japanese quail model, we have shown for the first time many unfavorable effects of the bee pollen supplementation on bones properties. Our results demonstrate that bee pollen supplementation (1.0% in feed) caused significant negative effects on the mechanical endurance of the tibia of quails in a sex-dependent manner, while showed beneficial effects on trabecular bone. Given the fact that males and females differ in bone metabolism and bee pollen may interact with sex hormones further research on mature animals should be performed to check if bee pollen could exert anti-osteopenic or anti-osteoporotic properties.
